# The Fiber-Optic Rotational Seismograph—Laboratory Tests and Field Application

**DOI:** 10.3390/s19122699

**Published:** 2019-06-15

**Authors:** Leszek R. Jaroszewicz, Anna Kurzych, Zbigniew Krajewski, Michał Dudek, Jerzy K. Kowalski, Krzysztof P. Teisseyre

**Affiliations:** 1 Faculty of Advanced Technologies and Chemistry, Military University of Technology, Warsaw 00-908, Poland; anna.kurzych@wat.edu.pl (A.K.); zbigniew.krajewski@wat.edu.pl (Z.K.); michal.dudek@wat.edu.pl (M.D.); 2 Elproma Electronics Ltd., Lomianki 05-092, Poland; j.kowalski@elpromaelectronics.com; 3 Institute of Geophysics, Polish Academy of Science, Warsaw 01-452, Poland; kt@igf.edu.pl

**Keywords:** rotational seismograph, fiber-optic sensor, rotational events, seismology, rotational seismology

## Abstract

The paper presents construction and laboratory tests, as well as the first field application of a new fiber-optic rotational seismograph. The system is based on a fiber-optic gyroscope (FOG), with determined Angle Random Walk of the order of 10^−8^ rad/Sqrt(s) and a few rad/s maximum detectable amplitude of rotation in the frequency range from direct current (DC) to 328.12 Hz. It has been designed for the rotational seismology area of interest. This work also presents exemplary relevant measurements, which were conducted using a set of two devices installed in the geophysical observatory in Książ, Poland.

## 1. Introduction

This paper deals with an innovative sensor suitable for rotational seismology, which falls within rotational ground movements from earthquakes, explosions, and ambient vibrations [1]. These motions are interesting for several reasons and can also provide additional constraints on the seismic source [2,3]. For the above reasons, it is interesting to a wide range of geophysical disciplines, including broadband seismology, strong-motion seismology, earthquake engineering, seismic hazards, earthquake physics, seismic instrumentation, seismotectonics, and geodesy, as well as to physicists connected with the Laser Interferometer Gravitational-Wave Observatory (LIGO) project. The practical aspect of the first three from the above disciplines might also have some effect on rocking and torsion, even accidental torsion, of engineering construction, as well as on distortion of high or long structures [4].

In spite of the growing popularity of rotational seismology, there is still lack of appropriate rotational sensors for its field application, also in the form of a seismograph which contains a rotational sensor, data acquisition system with precise sensor localization and precision time monitoring. One can distinguish several technologies of rotational sensors, starting from mechanical systems basing on pendulum seismometers [5] or geophones [6,7], through micro-electro-mechanical system (MEMS) gyro [8], up to ring laser [9] and fiber-optic gyros [5,10]. Nevertheless, rotational sensors used in field application should meet some technical requirements forced by the rotational seismology, which one can find in the paper [11], where the comparison of above listed different solutions is also made. However, applications of the above rotational sensors are generally used separately, and for this reason, the reliability of the recorded data can be controversial. The comparison of results obtained by different devices concerning the same rotational events can be found in limited papers, for instance [5,11,12,13]. In order to gather reliable data, the idea of application of at least two systems designed according to the same technology, like for instance, in the well-known paper [14], is interesting and applied in this paper.

For the above reasons, this extended paper relates to a manuscript presented at the 7th International Symposium on Sensor Science (I3S2019) in Naples, Italy [15], in which we described the construction and laboratory tests, as well as results of field application of the set of two identical FOSREM^®^—the innovative Fiber-Optic Rotational Seismograph. During the field test conducted in the geophysical observatory in Książ, Poland, torsion and tilt effects resulting from mining seismic quakes induced by copper mining operations have been recorded by them (FOSREMs) with high accuracy.

## 2. Construction and Laboratory Investigation of the Fiber-Optic Rotational Seismograph—FOSREM^®^

### 2.1. FOSREM^®^ Construction

**Figure 1:**
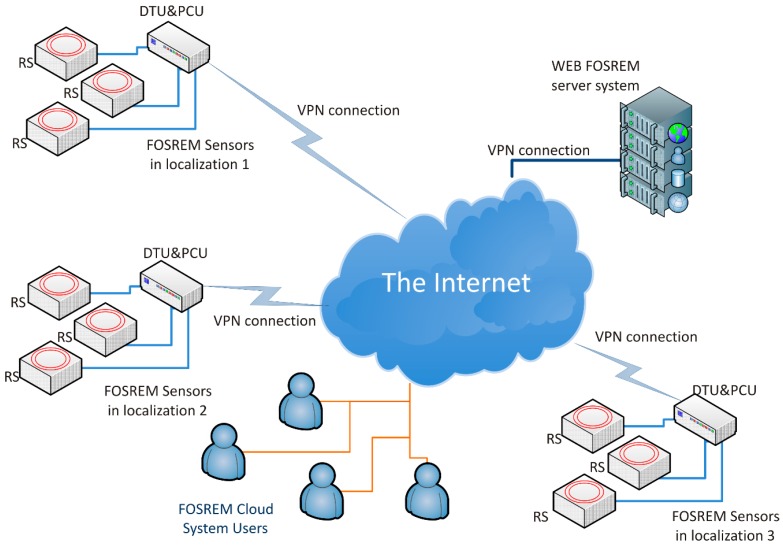
The Fiber-Optic Rotational Seismograph (FOSREM^®^) cloud system with samples FOSREM Sensors localization [16]. DTU: data transmission units. PCU: power communication units. VPN: virtual private network.

FOSREM^®^, based on a fiber-optic rotational seismometer, operates as a one-axis rotational sensor. A dozen FOSREM sensors can operate in one worldwide network (Figure 1), transferring data to a central cloud-based server system—WEB FOSREM—used for data storage, monitoring the sensors’ work, as well as for the remote control of their parameters [16]. This approach protects data to view and analyze from anywhere in the world via the Internet. At a given localization, FOSREM^®^ contains two main parts: FOSREM sensors—up to three—and DTU and PCU—data transmission, and power communication units. Regarding the description in Ref. [13], DTU enables a synchronous data recording from sensors with collecting information on a local disc, as well as transferring it to PCU via fiber link. PCU enables future data transmission using the internet or GSM/GPS to WEB FOSREM with a rate of up to 100 Mbps, as well as the power supply of sensors and DTU. Since in the rotational seismology the rotational event exists as sudden changes, each FOSREM sensor has been constructed by applying a minimum configuration of the fiber-optic gyroscope (FOG) [17], where the Sagnac effect [18] produces a phase shift (∆*φ*) between two counter-propagating light beams proportional to a measured rotation rate (Ω) [17]:

Ω = *S*_o_ ∆*φ* = (λc/4π*RL*)∆*φ,* (1)

where *S*_o_ is the optical constant of the system, *λ* is the wavelength of the used light source, *c* is the velocity of light in the vacuum, *L* is the length of fiber in the sensor loop, and *R* is the sensor loop radius.

**Figure 2:**
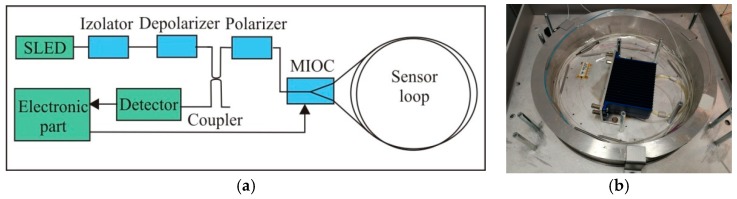
The optical part of the sensor according to a minimum gyro configuration: (**a**) block diagram, (**b**) technical realization for FOSREM-1. MIOC: multifunction integrated optic chip. SLED: superluminescent light emitting diode.

The main advantages of such a solution is its practical sensor insensitivity to linear motions and direct measurement of a rotational rate. Physically each sensor can be divided into two basic parts: optical and electronic. The optical part, according to the schema in Figure 2, assures the Sagnac effect reversibility [17] and is constructed according to the description presented in our previous papers [11,13,15,19]. Only a substitution of a fiber coupler in loop and a piezoceramic phase modulator by a multifunction integrated optic chip (MIOC) (Idealphotonics Ltd., Shanghai, China) have been applied. The LiNbO_3_ MIOC structure has Y junction with single-polarization proton-exchange waveguides and a pair of push–pull electrodes. It protects a good selection of linear polarization for an input–output way, as well as a proper phase modulation with a flat efficiency over a large bandwidth. Such elements protect the processing scheme, called serrodyne modulation, which uses a linear phase ramp first proposed by Ardity et al. [20]. Based on noise investigation [10], the optical part uses a 5000 m optical fiber in a sensor loop with a radius of 0.1075 m, which used an InGaAs PIN photodiode (Optoway Technology Ltd., Hsin Hu - Taiwan) as a detector and total optical losses of about 20 dB protect sensitivity of the order of 10^−8^ rad/s/√Hz [19]. The standard single-mode fiber SMF-28e+ (Corning Inc., New York - USA) with attenuation of 0.322 dB/km was wound using a special kind of winding named a double-quadrupole mode, according to minimalization of the thermal Shupe effect [21]. The used continuous broadband superluminescent light emitting diode (SLED) (Exalos AG, Schlieren - Switzerland) with a bandwidth of 37.9 nm, a central wavelength of 1313.1 nm, and an optical power of 10 mW leads to depolarization of beams propagating in the loop [19]. This approach ensures the elimination of the polarization effect of interacting beams on the output signal [22].

**Figure 3:**
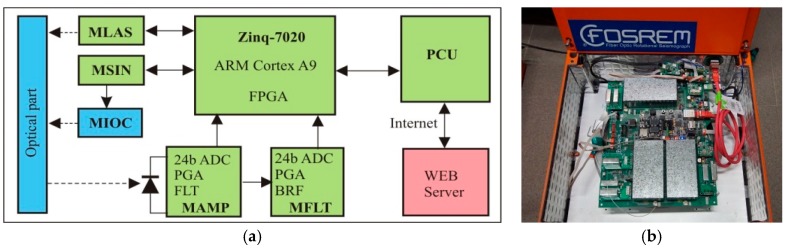
The electronic part of the sensor: (**a**) block diagram, (**b**) technical realization for FOSREM-1. MLAS: module of SLED controller, MSIN: module of sine signal generation, MIOC: module of signal generator for MIOC, MAMP: module of amplifier, MFLT: module of filter, FPGA: field programmable gate array, PGA: prograsmmable gain amplifier, FLT: low-pass filter, BRF: band-rejected filter.

Calculation of the detected rotation is based on the following equation [13]:

Ω = *S*_o_ arctan[*S*_e_*u*(*t*)] = *S*_o_ arctan[*S*_e_ (*A*_1__ω_/*A*_2__ω_)], (2)

where: *A*_1ω_, *A*_2ω_ are the first and second amplitudes of the harmonic output signal *u*(*t*), *S*_e_ is the electrical constant related to parameters of applied components. The above calculation is created by the sensor’s electronic part, which performs synchronous detection in a digital form with a special procedure of selecting and amplification of the first and second amplitude of harmonic output signal, due to a large difference (4–5 orders of magnitude) of their amplitudes [13]. As one can see in Figure 3, the hardware of this part contains digital units with sophisticated software for system control and real-time data computation. The main element is the Z-turn board (*XC7Z020-1CLG400C, MYIR*), based on the Xilinx Zynq-7000 all programmable system-on-chip (SoC). It integrates a dual-core ARM Cortex-A9-based processing system and a 28 nm Xilinx programmable logic in a single device. The Z-turn board allows for communication by 10/100/1000 Ethernet and USB. All electronic modules described below as moldule of amplifier (MAMP); module of filter (MFLT); module of signal generator for MIOC (MIOC); module of SLED controller (MLAS) are connected to IP core running on the field programmable gate array (FPGA) using a hardware interface. The MAMP used for amplification of the detected signal is connected with a photodiode and contains a trans-impedance amplifier, low-pass filter (FLT), programmable gain amplifier (PGA), and 24-bits 1.5 Msps analog-to-digital converter (ADC). The MFLT protects filtration of the first and second harmonic output signal and contains a special digitally controlled band-rejected filter (BRF), FLT, PGA, and above described ADC. Analog signals in each channel are simultaneously sampled by each ADC connected to the FPGA chip. Applied analog parts define high accurate acquisition with almost 1Msps sampling and over 100 dB dynamic range. The MSIN protects the suitable modulation analog signal for the MIOC, which 16-bit digital-to-analog converter (DAC) connected to the FPGA chip works simultaneously with ADCs. Finally, the MLAS services the control and state signal of the light source SLED. The dedicated IP core collects raw data from ADCs and transfers it to the ARM Cortex-A9 processor, which performs all computation including phase wrapping (continuously phase counting above 2π). It also performs communication transfer to the communication unit or the Internet. The connection provides data transmission and power supply over a single ethernet cable within a distance of 100 m.

**Figure 4:**
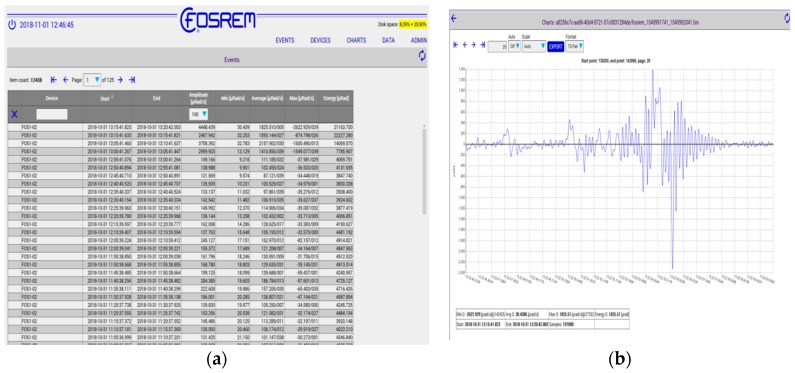
Print Screen for WEB FOSREM: (**a**) screen with selected and stored system events, (**b**) example of recorded seismic torsion event.

Finally, the obtained results are stored on a hard disc and transmitted to the mentioned previously telemetric server (WEB FOSREM), which additionally can be used for remote control for all sensors (see Figure 4).

The remote control, the possibility of the independent power supply, and the relatively small dimension of the rotational sensor (360 × 360 × 180 mm) and their weight ~10 kg makes it a fully mobile device.

### 2.2. Results of Laboratory Investigation

**Figure 5:**
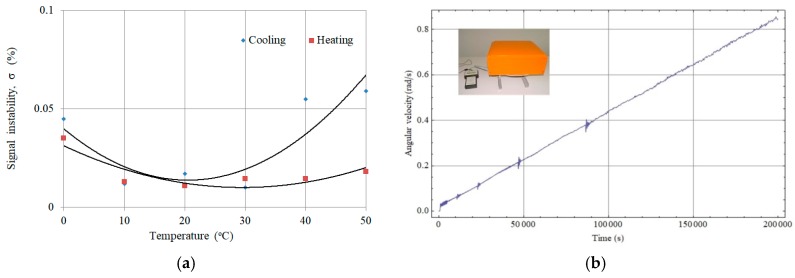
Example of a laboratory test of FOSREM-2: (**a**) temperature stability for the cooling and heating cycle [19], (**b**) recording rotational speed with increasing angular velocity up to 0.9 rad/s.

The proper FOSREMs’ work required in the first step their calibration by determination of optical and electrical constants (*S*_o,_ *S*_e_). It was made on the basis of the Earth rotation measurement, according to the procedure described in detail in our previous paper [10]. Several experimental tests were carried out to confirm FOSREMs’ parameters and reliability, including experimental uncertainties calculation based on the registration of the rotational component of the Earth at a different frequency bandpass [19], recording strong rotation motion with a new setup using earthquake simulation [11], and other. As an example, in Figure 5a a thermal test in a climate chamber VCL 7010 (Votsch Industrietechnik, Balingen-Frommern - Germany) at the temperature range of 0–50 °C for FOSREM-2 is presented [19]. As one can see, the recorded thermal instability of an output signal is less than 0.06%/°C, including the cooling and heating cycle. Similarly, we obtained a good linearity for FOSREMs regarding detection rotation with a high angular velocity up to radian per second (limitation of used measurement equipment), which is presented in Figure 5b. The observed perturbation for an angular velocity of around 0.05, 0.10, 0.22, and 0.38 rad/s is connected with the resonant characteristics of the rotation table.

**Figure 6:**
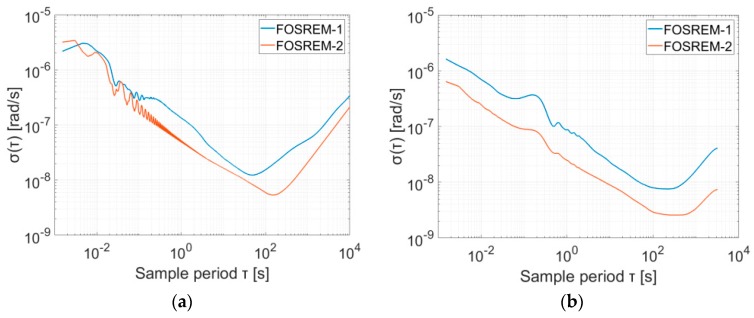
The Allan variance analysis for FOSREM-1 and -2 determined from the data gathered in various positions of the FOSREMs: (**a**) sensors were placed on the sturdy flat floor in the basement of the laboratory; (**b**) sensors were placed on the active optical table.

Finally, regarding the investigation of the system sensitivity, as well as its drift, the Allan variance analysis (AV) has been performed [23,24]. Figure 6 presents the results of these analyses for FOSREM-1 and -2. The data for AV were gathred at MUT in Warsaw, Poland for two positions of the FOSREM: on a sturdy flat floor in the basement of the laboratory and on an active optical table (Thorlabs, Newton - USA). Moreover, the data were recorded in nightly hours to reduce urban noise. The plots presented in Figure 6 point out that the appropriate method of error estimating is significant, but environmental conditions are crucial and they can provide false results. The parameters of ARW (Angle Random Walk) and BI (Bias Instability) determined from data gathered when sensors were placed on the active optical table were more reliable. The plot in Figure 6a is more disturbed, and the values of ARW and BI based on this plot have a higher value (Table 1). The disturbances in Figure 6b are results of resonance characteristics of the applied table, nevertheless, it indicates lower values of ARW and BI; ARW is equal to 8.66 × 10^−8^ rad/√s and 2.45 × 10^−8^ rad/√s, whereas BI has a level of 1.13 × 10^−8^ rad/s and 3.91 × 10^−9^ rad/s for FOSREM-1 and -2, respectively.

The obtained value of ARW is in good correlation with the theoretical sensitivity of FOSREM mentioned in Section 2.1. Taking into account the total losses of the optical part (24.68 dB and 19.02 dB for FOSREM-1 and -2, respectively), the sensitivity equals 6.00 × 10^−8^ rad/s/√Hz and 2.83 × 10^−8^ rad/s/√Hz has been expected for FOSREM-1 and -2, respectively.

Since the determined parameters fulfill all requirements described for rotational seismology [11], so it can be concluded that two sensors used as FOSREM seem to be appropriate for registration of rotational events associated with rotational seismology.

## 3. FOSREM^®^ in the Field Application

**Figure 7:**
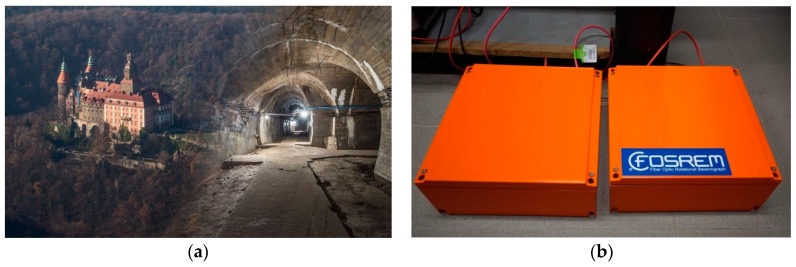
FOSREMs’ field application: (**a**) Książ castle with view of the underground tunnel located below the surface of the castle, (**b**) mounted devices in the geophysical observatory.

**Figure 8:**
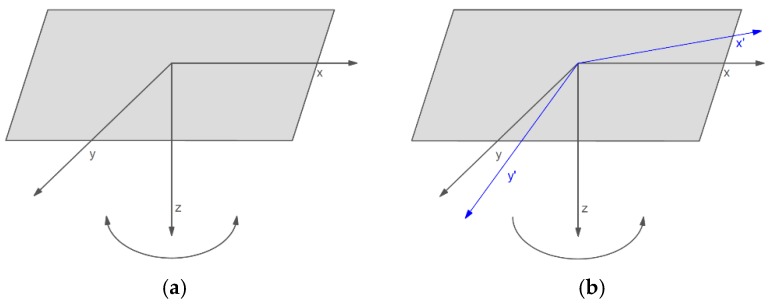
The rotational events recorded by FOSREMs: (**a**) torsion, (**b**) tilt.

The set of two FOSREMs (recognized by WEB FOSREM as FOSREM-1 and FOSREM-2) has been mounted in the geophysical observatory of the Polish Academy of Science in Książ, Poland, which is located in the area of mining activity (position *φ* = 50°50′31″ N, *λ* = 16°17′29″ E). The seismometers have been installed on a special pedestal mounted horizontally for seismological measurement located at a depth of about 49 m below the main castle courtyard next to each other (Figure 7). In this position, the FOSREMs can detect two kinds of rotation events around vertical direction only, named torsion and tilt, as is shown in Figure 8. The first of them, the torsion (Figure 8a) is a rotational component around a vertical axis recorded by FOSREM. Physically, the torsion can be treated as the pedestal oscillations around vertical axes with variable amplitude, after that the pedestal returns to the previous position. The second one, the tilt is often misunderstood or unclear and there are many definitions of it. In this paper, the tilt, physically, is an angle rotating in one direction around a vertical axis (Figure 8b) caused by crumps. The crumps are a dynamic phenomenon caused by a rock mass shock, as a result of which an excavation appears or its part is destroyed or damaged very suddenly at local mines. The above rotational events were recorded by FOSREMs at a frequency band between 0 Hz to 10.25 Hz.

The obtained data [stored at WEB FOSREM as events—see example in Figure 4b] were analyzed in a specially designed Matlab script, which allows us to read the data from both FOSREMs simultaneously and to calculate the Pearson’s correlation coefficient (*P*_C_) between the signals from FOSREMs according to the following formula [25]:

*P_c_* = *cov*(*x*, *y*)/(*σ*_x_, *σ*_y_), (3)

where: *cov*(*x*, *y*) is the covariance between variables *x* and *y*, *σ*_l_ is the standard deviation in the population *l*.

**Figure 9:**
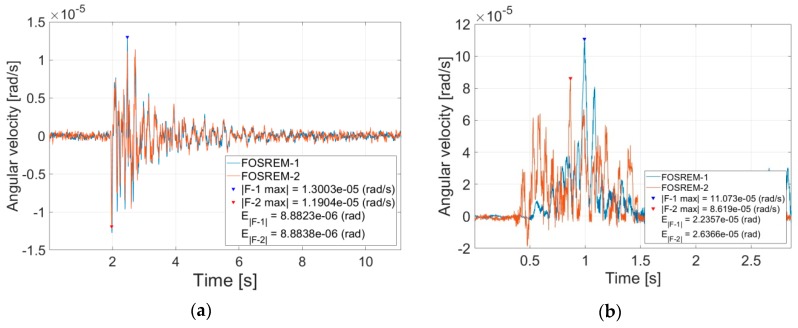
The example of data recorded by the FOSREMs in the geophysical observatory in Książ, Poland: (**a**) torsion event on the 1 December 2017, at 12:19 UTC with *P*_C_ = 0.98, (**b**) tilt event on the 13 December 2017, at 11:15 UTC with *P*_C_ = 0.57.

Figure 9 presents the exemplary data of the recorded torsion and tilt by two installed devices. Every figure also shows information about the absolute value of the signal maximal amplitude (|*F*-1 max|, |*F*-2 max|), as well as an *E*_F_—energy coefficient (*E*_|*F*-1|_, *E*_|*F*-2|_)—calculated numerically using a method of rectangles of the Riemann integral for FOSREM-1 and FOSREM-2, respectively.

In Table 2, we present a collection of the same recorded torsion and tilt events in a period between 29 August 2017 and 3 February 2018. There is information about a recorded by FOSREMs maximal signal amplitude, energy coefficient (*E*_F_), as well as the correlation coefficient (*P*_C_) between FOSREM-1 and FOSREM-2. As mentioned above, the energy coefficient *E*_F_ of tilt can be physically, directly indicated with a value of pedestal angle rotating in one direction around a vertical axis which is in the range of a few to hundred microradians.

One can see that torsion recording is characterized by a much higher value of the *P*_C_ (average value equal to 0.96 ± 0.03) between the FOSREMs than for tilt recordings (average value equal to 0.63 ± 0.06). We concluded that high correlation of torsion events recorded by FOSREM-1 and FOSREM-2 indicated a good compatibility of the FOSREMs’ signals, which could be treated as proof of their usefulness for recording rotational events. On the other hand, a much smaller correlation obtained for the recorded tilt is thought provoking. Our recording of a tilt phenomenon is probably one of the first recordings in this matter, and the obtained data analysis shows that we made a mistake during FOSREMs’ installation. The relative small average value of *P_C_* for them (0.63 ± 0.06) can be connected with the method of FOSREMs mounted on the seismological pedestal. The FOSREMs should have been stiff mounted to the pedestal despite their relatively high weight (about 10 kg), because the tilt is in the range of microradians only. As one can see from data presented in Table 1, the value of the maximal recorded amplitude (9.35 × 10^−4^ rad/s), as well as energy coefficient (1.17 × 10^−4^ rad) for tilt events is much higher than for recorded torsion events (3.25 × 10^−5^ rad/s; 1.91 × 10^−5^ rad). The nature of the tilt phenomenon, which is more rapid due to its source of generation, for example, caving, probably needs a better sensor protection against its movement.

## 4. Conclusions

The FOSREM^®^—Fiber-Optic Rotational Seismograph—presented in this paper, seems to be a suitable instrument for rotational seismology. It uses the mobile fiber-optic rotational seismometers, enabling it to detect rotational movements in a wide amplitude (from a few 1.13 × 10^−8^ rad/s up to a few rad/s), as well as in a suitable frequency range (from DC up to 328.12 Hz). Because the FOSREM^®^ is fully remote controlled by the internet, it is suited for autonomous work in a very long period. Thus, it is useful for a systematic seismological investigation at any place.

The collected data in the geophysical observatory in Książ, Poland resulted from mining seismic quakes induced by copper mining operations. Their recording by a set of two FOSREMs showed a very high correlation coefficient between the applied sensors regarding torsion events, which confirms the records’ reliability. The average Pearson’s correlation coefficient in the range of 0.96 confirms FOSREM^®^ usefulness for such investigation. The data connected with tilt events were not so well correlated, but it was caused by a mistake connected with FOSREMs’ installation at the seismological pedestal. In future research, the careful attention at the FOSREMs’ stiff mounting to the pedestal should be made. However, the collected data indicated the rapid nature of tilt phenomenon, which is reflected in the higher value of the recorded signal amplitude than in the case of torsion recordings. To the authors’ knowledge, presented in this paper recordings of tilt effects caused by crumps at local mines are unique of the carried-out research in the world. FOSREM^®^ gives great opportunities for spreading knowledge about seismic rotational events, as well as torsional effects existing in any engineering constructions. According to the above, in the author’s opinion, the presented FOSREM^®^ is appreciated for creating the growing interest in rotational seismology by providing significant data.

## Figures and Tables

**Table 1:** The parameters of Angle Random Walk (ARW) and Bias Instability (BI) for FOSREM-1, -2 determined from the data gathered in various positions of FOSREMs.

	Sturdy Flat Floor		Active Optical Table	
	ARW [rad/√s]	BI [rad/s]	ARW [rad/√s]	BI [rad/s]
FOSREM-1	1.33 × 10^−7^	1.81 × 10^−8^	8.66 × 10^−8^	1.13 × 10^−8^
FOSREM-2	5.26 × 10^−8^	8.08 × 10^−9^	2.45 × 10^−8^	3.91 × 10^−9^

**Table 2:** Data recorded by the FOSREMs in the geophysical observatory in Książ, Poland in a period of 29 August 2017–3 February 2018.

Device	Recorded Torsion Event					Recorded Tilt Event				
	Date	Time	Max. Amplitude [rad/s]	*E*_F_ [rad]	*P*_c_	Date	Time	Max. Amplitude [rad/s]	*E*_F_ [rad]	*P*_c_
FOSREM-1 FOSREM-2	29 August 17	11:02:34	2.68 × 10^−5^ 3.25 × 10^−5^	1.31 × 10^−5^ 1.91 × 10^−5^	0.99	8 December 17	13:01:41	9.61 × 10^−5^ 9.31 × 10^−5^	2.19 × 10^−5^ 2.20 × 10^−5^	0.71
FOSREM-1 FOSREM-2	29 August 17	11:08:12	1.23 × 10^−5^ 1.21 × 10^−5^	9.00 × 10^−6^ 9.12 × 10^−6^	0.98	13 December 17	11:15:27	1.11 × 10^−4^ 8.62 × 10^−5^	2.34 × 10^−5^ 2.64 × 10^−5^	0.57
FOSREM-1 FOSREM-2	1 December 17	12:19:10	1.30 × 10^−5^ 1.19 × 10^−5^	8.88 × 10^−6^ 8.88 × 10^−6^	0.98	11 January 18	11:27:05	7.97 × 10^−6^ 8.46 × 10^−6^	1.89 × 10^−6^ 1.61 × 10^−6^	0.59
FOSREM-1 FOSREM-2	13 December 17	18:25:43	3.03 × 10^−6^ 3.26 × 10^−6^	2.22 × 10^−6^ 2.56 × 10^−6^	0.93	18 January 18	9:44:34	5.21 × 10^−5^ 5.47 × 10^−5^	1.52 × 10^−5^ 1.23 × 10^−5^	0.59
FOSREM-1 FOSREM-2	13 December 17	18:25:58	1.78 × 10^−6^ 2.08 × 10^−6^	1.18 × 10^−6^ 1.52 × 10^−6^	0.92	25 January 18	11:55:33	9.35 × 10^−4^ 8.76 × 10^−4^	1.03 × 10^−4^ 1.17 × 10^−4^	0.67
FOSREM-1 FOSREM-2	14 December 17	08:06:24	6.04 × 10^−6^ 6.13 × 10^−6^	4.26 × 10^−6^ 4.80 × 10^−6^	0.97	26 January 18	11:11:18	2.34 × 10^−4^ 2.21 × 10^−4^	3.77 × 10^−5^ 3.78 × 10^−5^	0.60
FOSREM-1 FOSREM-2	8 January 18	08:09:02	3.88 × 10^−6^ 3.44 × 10^−6^	4.43 × 10^−6^ 4.66 × 10^−6^	0.95	26 January 18	11:11:43	4.99 × 10^−4^ 6.61 × 10^−4^	4.92 × 10^−5^ 5.82 × 10^−5^	0.57
FOSREM-1 FOSREM-2	8 January 18	08:09:57	1.18 × 10^−5^ 1.29 × 10^−5^	9.31 × 10^−6^ 9.56 × 10^−6^	0.98	26 January 18	11:11:58	4.98 × 10^−4^ 4.78 × 10^−4^	5.76 × 10^−5^ 4.95 × 10^−5^	0.73
FOSREM-1 FOSREM-2	26 January 18	11:14:23	1.42 × 10^−5^ 1.40 × 10^−5^	1.22 × 10^−5^ 1.21 × 10^−5^	0.97	3 February 18	10:14:21	2.40 × 10^−4^ 2.85 × 10^−4^	4.25 × 10^−5^ 5.64 × 10^−5^	0.61
**Average value of *P*_c_ for torsion event**				**0.96 ± 0.03**		**Average value of *P*_c_ for tilt event**			**0.63 ± 0.06**	
